# Limonin induces apoptosis of HL‐60 cells by inhibiting NQO1 activity

**DOI:** 10.1002/fsn3.2109

**Published:** 2021-03-02

**Authors:** Yunyi Chen, Jiaojiao Liang, Xiao Liang, Jiebiao Chen, Yue Wang, Jinping Cao, Chongde Sun, Jiaming Ye, Qingjun Chen

**Affiliations:** ^1^ Laboratory of Fruit Quality Biology/The State Agriculture Ministry Laboratory of Horticultural Plant Growth, Development and Quality Improvement Zhejiang University Hangzhou China; ^2^ Zanyu Technology Qingshan Lake Science and Technology City Hangzhou China

**Keywords:** antioxidant enzymes, cancer, cell apoptosis, limonin, NQO1

## Abstract

Limonin is an important bioactive substance in citrus fruits, especially in seeds, which has great potential in cancer prevention and treatment. In order to explore the anticancer activity based on interaction between limonin and NQO1, Human promyelocytic leukemia cells (HL‐60) were studied in vitro. We found that limonin could inhibit proliferation and promote apoptosis of HL‐60 cells, and the effect was positively correlated with its dosage. Western blot results showed that limonin could activate the endogenous apoptosis pathway mediated by mitochondria via up‐regulating pro‐apoptotic proteins (Bax, cytochrome c, Caspase3, and Caspase9) and down‐regulating anti‐apoptotic proteins (Bcl‐2), thus inhibiting the proliferation of HL‐60 cells and promoting apoptosis, which further proved the anticancer activity of limonin from the molecular mechanism. At the same time, limonin down‐regulated the expression of NQO1, indicating that limonin may indirectly act on the apoptosis pathway by regulating the expression activity of antioxidant enzymes in vivo, thus exerting its inhibitory effect on tumor cells, which provides an idea for the molecular mechanism that natural products can indirectly exert their anticancer effect by regulating the activity of antioxidant enzymes.

## Graphical abstract

Limonin, a citrus nutrient factor, was used as the subject material, which showed a proliferation inhibitory effect on HL‐60 cells. Mechanism exploration found that this effect was caused by activating the apoptosis pathway through inhibiting NQO1 expression.

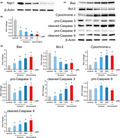

## 1 INTRODUCTION

*Citrus reticulate* Blanco (Rutaceae, Aurantinoideae) is one of the most important economic fruit which is widely cultivated in tropical and subtropical regions (Wang et al., 2017). Citrus is highly favored because of its colorful appearance, pleasant aroma, and sweet–sour taste. Citrus fruits are rich in nutrients, in addition to some common nutrients, such as vitamins and organic acids, citrus fruits also contain some unique bioactive substances, such as flavonoids, limonoids, carotenoids, coumarins, and volatile substances (Zhang et al., 2014). Mounting studies have shown that these natural citrus products exhibit a variety of biological activities including anticancer, anti‐inflammatory, and blood glucose regulation. (Dhiman et al., 2012; Liu et al., 2020; Manassero et al., 2013; Wang, Ji, et al., 2019; Wang et al., 2019). Among the biological activities of citrus, chemoprophylaxis against cancer has always been a research hotspot. Due to the characteristics of citrus natural products such as easy availability and low toxic, researchers hope to use citrus‐derived substances to inhibit tumor development (Tian et al., 2001) or mitigate the side effects (Tahaghoghi‐Hajghorbani et al., 2019)of chemotherapy drugs.

Cancer is one of the most important leading causes of death. Cancer causes great psychological and physical damage to people, especially some adolescent cancers, such as juvenile leukemia, will be devastating to the life will and family hope when the blow. Cancer prevention, in addition to avoiding exposure to carcinogens and keeping regular exercise, supplementing with natural products, especially fruits, is also a very important measure (Filocamo et al., 2015).

**FIGURE 1:**
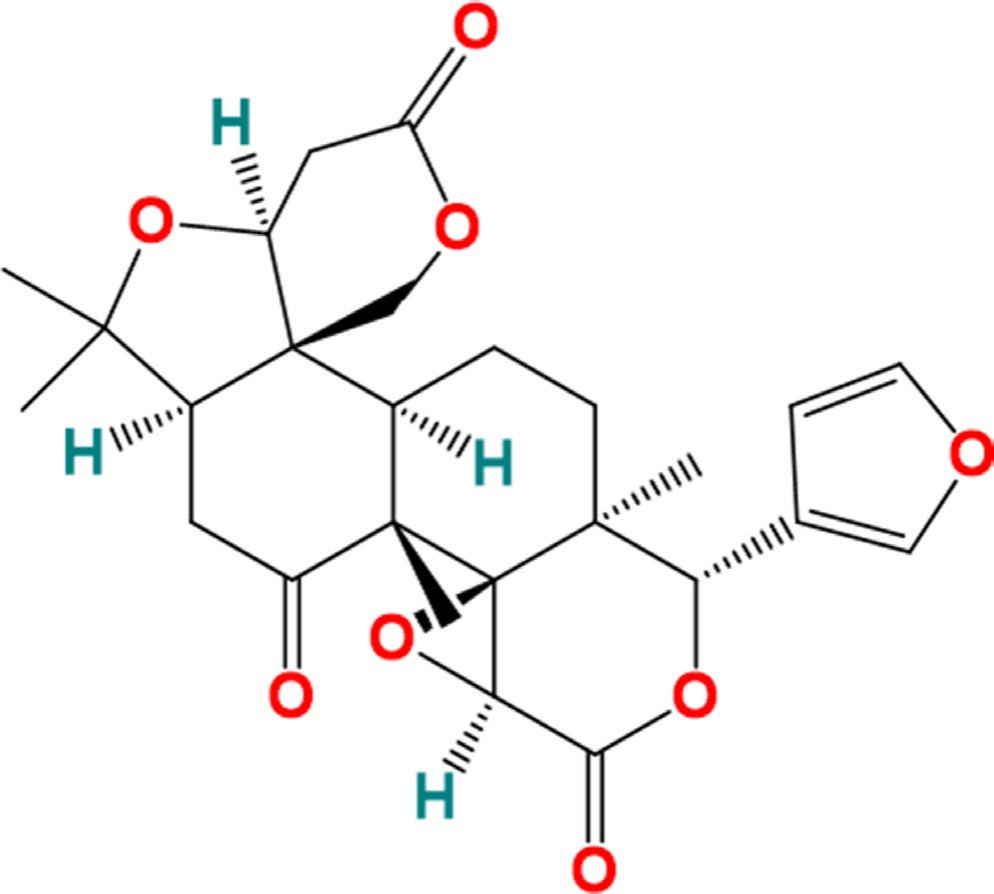
The chemical structure of limonin

Limonin (Figure 1), also known as obaculactone and evodin, is a type of secondary metabolites belonging to the tetracyclic triterpenoids, which is usually found in the plants of Rutaceae and Meliaceae. Limonin is extensively distributed in citrus fruits, which is the main cause of citrus bitterness (Emerson, 1948). Limonin has broad‐spectrum biological activities, such as anticancer, antiviral, antioxidant, antimicrobial, anti‐inflammatory, anti‐obesity, liver protection, and anti‐atherosclerosis (Higby, 1938, Emerson, 1948, Kelley et al., 2015; Lee et al., 2016; Yang et al., 2014). Rahman et al. (2015) found the anticancer effect of limonin on various human cancer cells by MTT method. Their results showed that limonin could significantly inhibit the growth of cancer cells by promoting the expression of apoptosis‐related proteins, and the effect was dose dependent. However, it has not been reported whether limonin could inhibit cancer by regulating the activity of antioxidant enzymes.

NAD (P)H Quinone Dehydrogenase 1 (NQO1) is an intracellular, cytosolic enzyme which is known to catalyze two‐electron reduction of a broad range of substrates. It plays an important role in the detoxification of environmental carcinogenic factors (Ross & Siegel, 2004). However, although NQO1 protects cells from oxidative stress (Davies et al., 2005), it also has carcinogenic effect inversely. Studies have shown that the expression of NQO1 is up‐regulated in many malignant tumors, such as melanoma, pancreatic cancer, intrahepatic cholangiocarcinoma, lung cancer, and breast cancer (Hu et al., 1996; Kelsey et al., 1997; Pan et al., 1995; Ross & Siegel, 2004). The overexpression of NQO1 can induce cell apoptosis and promote the proliferation of cancer cells (Luo et al., 2018). At present, evidence has shown that natural products can exert anticancer activity through inhibiting the expression of NQO1 (D'Anneo et al., 2010; Pink et al., 2000; Zhao & Wu, 2018), but there are few reports on the similar function of active substances in citrus fruits, especially limonin.

Our present study found that limonin can promote the apoptosis of promyelocytic leukemic cell line (HL‐60) by inhibiting the activity of NQO1, which provides new evidence and support for the exploration of the bioactivity of limonin and the regulation function of natural products on antioxidant enzyme activity.

## 2 MATERIALS AND METHODS

### 2.1 Materials and reagents

Limonin standard, dimethyl sulfoxide (DMSO), phosphate‐buffered saline (PBS), dicoumarol, acetonitrile, and methanol of chromatographic grade were purchased from Sigma‐Aldrich (St. Louis, MO, USA). Culture medium RPMI‐1640, fetal bovine serum (FBS), N‐2‐hydroxyethylpiperazine‐N‐2‐ethane sulfonic acid (HEPES), trypsin‐EDTA, and penicillin–streptomycin were purchased from Gibco (Waltham, MA, USA). Cell counting kit‐8 (cck‐8) was purchased from Dojindo Technologies, Inc. (Shanghai, China). NQO1 activity Assay Kit was purchased from Abcam (Cambridge, MA, USA). Enhanced BCA Protein Assay Kit, Annexin V‐FITC, and propidium iodide (PI) were purchased from Beyotime Institute of Biotechnology (Hangzhou, China). NP40 Lysis Buffer, Halt™ Protease and Phosphatase Inhibitor Cocktail, PVDF membrane (0.45 μm) were purchased from ThermoFisher Scientific (Shanghai, China). Sodium dodecyl sulfate‐polyacrylamide gel electrophoresis (SDS‐PAGE) gels were purchased from Genscript (Hangzhou, China). Electrogenerated chemiluminescence (ECL) kit was purchased from Service Bio (Wuhan, China). NQO1 antibody, Bax antibody, Bcl‐2 antibody, cytochrome c antibody, pro‐Caspase 3 antibody, cleaved‐Caspase 3 antibody, pro‐Caspase 9 antibody, cleaved‐Caspase 9 antibody, and β‐actin were purchased from Proteintech (Rosemont, IL, USA). Double distilled water was used in all experiments. All solutions for HPLC and LC‐MS analysis were filtered through a 0.22 μm membrane before injection. All other reagents and solvents of analytical grade were purchased from Sinopharm Chemical Reagent Co., Ltd. (Shanghai, China).

### 2.2 Cell line and cell culture

Human promyelocytic leukemia cells (HL‐60) were purchased from Shanghai Institute of Biochemistry and Cell Biology, Chinese Academy of Sciences. The cells were cultured in RPMI 1640 medium supplemented with 10% FBS, 20 mM HEPES, 100 U/ml penicillin, and streptomycin. Cells were maintained at 37°C in a humidified incubator containing 5% CO_2_. Exponentially growing cells were used for further experiments.

### 2.3 Cell viability assay

Cell viability of HL‐6 cells was evaluated by a cck‐8 assay according to Wang, Zang, et al. (2019) with small modifications. Briefly, cells at exponentially growing phase were seeded in flat bottomed 96‐well plates at an initial density of 1 × 10^4^ cells/well with 200 μl medium. Limonin and dicoumarol were dissolved in DMSO (DMSO accounted for 2.0% of the final volume, in the ratio of which the limonin solubility was 94.19%, and exerted no significant threat to the cell viability based on our pre‐experiment). Cells were treated with serial dilutions of limonin (25, 50, 100, 200, and 400 μM). Dicoumarol with a concentration of 5 μM was set as positive control, and DMSO was set as solvent control. After incubation for 24 hr, the medium was removed and the cells were washed twice with PBS. Cck‐8 reagents diluted with FBS‐free RPMI 1640 medium was added to the wells and incubated for 1h. The absorbance was detected at 450 and 620 nm by a microplate reader (Synergy H1, BioTek, Winooski, VT, USA). The cell viability was calculated as follows: Cell viability (%) = Treatment (A_450_–A_620_)/Solvent control(A_450_–A_620_) × 100%. Each experiment was performed in triplicate and repeated three times independently.

### 2.4 Cell apoptosis assay

Cells were treated with serially diluted limonin (50, 100, and 200 μM). Dicoumarol with a concentration of 5 μM was set as positive control, and DMSO was used as solvent control. After treatment, the cells were washed twice with ice‐cold PBS. Trypsin (0.25%) / EDTA (0.02%) was applied to dissociated the adherent cells. Both suspension and attached cells were collected after centrifuging at 85 g for 5 min, and the density was adjusted to 1 × 10^3^ cells/ml. Apoptotic cells were identified by double supravital staining. For 1 ml cells, 200 μl of Annexin V‐FITC was added to the cells followed by the addition of 10 μl of propidium iodide (PI). The samples were stained for 5 min in the dark at room temperature. Then cell apoptosis rate was detected immediately by flow cytometry.

### 2.5 Quantitative Real‐Time PCR assay

The Quantitative Real‐Time Polymerase Chain Reaction (qRT‐PCR) was performed according to our previous report with small modifications (Wang et al., 2020). Briefly, cells were plated in 6‐well plates with densities of 2 × 10^6^ cells per well for HL‐60. After 24 hr of culture, the cells were treated with limonin at a concentration of 50–200 μM for 12 hr, respectively, 5 μM diucoumarol was used as positive control. Total RNA isolation, cDNA synthesizing, and Quantitative Real‐Time PCR were performed using commercial kits according to the manufacturer's protocols. The primer sequences were listed in Table 1. GAPDH was used as the control, and the relative level of gene expression was calculated using the 2^−ΔΔCt^ method. Each experiment was repeated three times independently.

**TABLE 1:** Sequences of primers used for qRT‐PCR detection

Genes	Sequences
*GAPDH*	F: TCA ACG GCA CAG TCA AGG R: ACT CCA CGA CAT ACT CAG C
*NQO1*	F: AAA GGA CAT CAC AGG TAA AC R: GGA ACT GGA ATA TCA CAA GG
*Bcl‐2*	F: TTT GAG TTC GGT GGG GTC AT R: TGA CTT CAC TTG TGG CCC AG
*Bax*	F: TGG CAG CTG ACA TGT TTT CTG AC R: TCA CCC AAC CAC CCT GGT CTT
*Caspase3*	F: GCA GCA AAC CTC AGG GAA AC R: TGT CGG CAT ACT GTT TCA GCA
*Caspase9*	F: GTG ACA TCT TTG TGT CCT AC R: CTG TTT ATA AAT CCC TTT CA

### 2.6 Western blot assay

The Western blot assay was conducted according to our previous study with small modifications. Briefly, HL‐60 cells were lysed by NP40 Lysis Buffer containing 1 × Halt™ Protease and a Phosphatase Inhibitor Cocktail, followed by vortex on ice using a cell crusher (JY98‐IIIDN, HUXI, Shanghai, China). The supernatant was harvested after centrifuging at 12,235 g for 10 min at 4°C. Protein concentration was determined by BCA Protein Assay Kit. Equal amounts of cell lysates were resolved on SDS‐PAGE gels and subsequently transferred into a PVDF membrane (0.45 μm). After incubation in a blocking solution containing with 5% (w/v) skim milk in tris‐buffered saline containing 0.1% Tween20 (TBST) at room temperature for 2 hr, the membranes were incubated with primary and secondary antibodies successively. The antibodies used in the study and their dilutions were listed as follows. Anti‐NQO1 (1:1,000), anti‐Bax (1:1,000), anti‐Bcl‐2 (1:1,000), anti‐Cytochrome (1:1,000), anti‐pro‐Caspase 3 (1:1,500), anti‐cleaved‐Caspase 3 (1:2,000), anti‐pro‐Caspase 9 (1:1,500), anti‐cleaved‐Caspase 9 (1:2,000), and anti‐β‐actin (1:5,000). β‐actin was used as the loading control. The blot complex was detected by an ECL kit using the ChemiDoc™ XRS+ System (Bio‐rad, Hercules, CA, USA). The amounts of protein relative to the control were quantified by Image Lab (Bio‐rad, Version 3.0, Hercules, CA, USA).

### 2.7 Statistics

All experiments were repeated at least three times, and data were expressed as the mean ± *SEM*. SPSS 19.0 software (IBM, Armonk, NY, USA) was used for statistical analyses. Significant differences among different groups were analyzed using one‐way ANOVA, followed by Tukey's test at *p* < .05. Graphical representations were performed with OriginPro (version 2019, OriginLab).

## 3 RESULTS

### 3.1 Inhibition activity of limonin on the proliferation of HL‐60 cell

**FIGURE 2:**
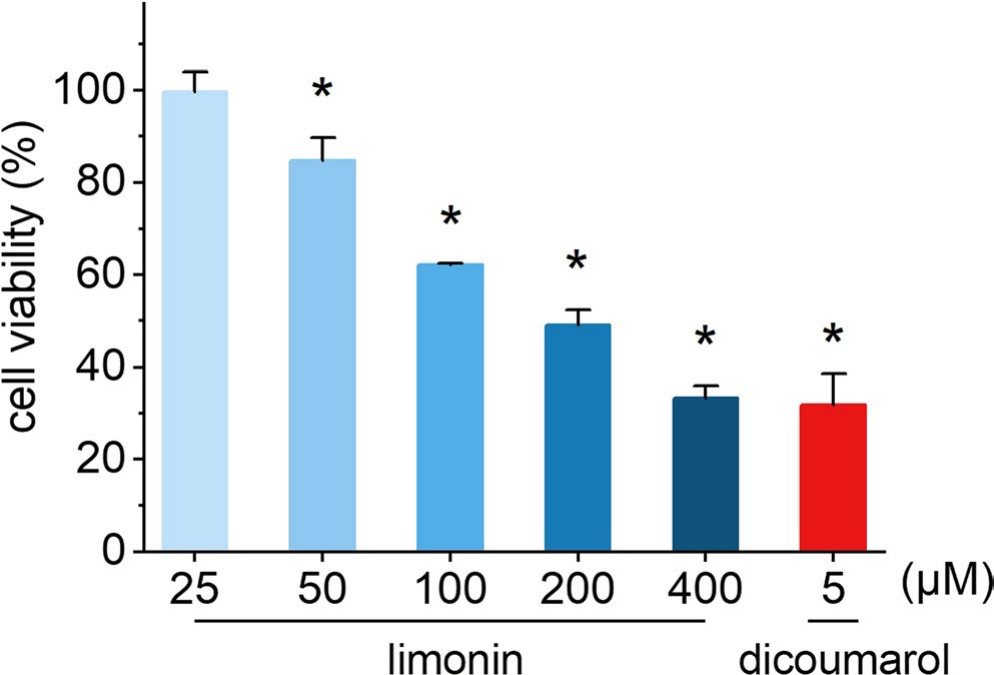
The effects of different concentrations of limonin on the proliferation of HL‐60 cells

To examine the effect of limonin on cell proliferation, HL‐6 cells were treated with various concentrations of limonin for 24 hr, and then, the cell viability was measured using a cell counting kit‐8 (cck‐8) assay. In this analysis, 5 μM dicoumarol (the specific inhibitor of NQO1) was used as positive control. The results demonstrated that 25 μM limonin exhibited the lowest cytotoxicity among five concentrations so far tested, with a cell viability close to 100% after 24 hr of incubation, while the positive control (5 μM dicoumarol) showed a strong activity of inhibiting the proliferation of HL‐60 cells. Compared with 25 μM limonin group, the cell viability of HL‐60 cells decreased significantly at the concentration of 50–400 μM in a dose‐dependent manner; in other words, the inhibition activity of limonin on cell proliferation was enhanced with the increase of concentration (Figure 2, *p* < .05). Notably, the suppression efficacy of 400 μM limonin was comparable with that of the positive control group. From these results, we can see that limonin showed inhibiting abilities of the proliferation of HL‐60 cells and its effect was positively correlated with its concentration.

### 3.2 Promotion activity of limonin on apoptosis of HL‐60 cells

**FIGURE 3:**
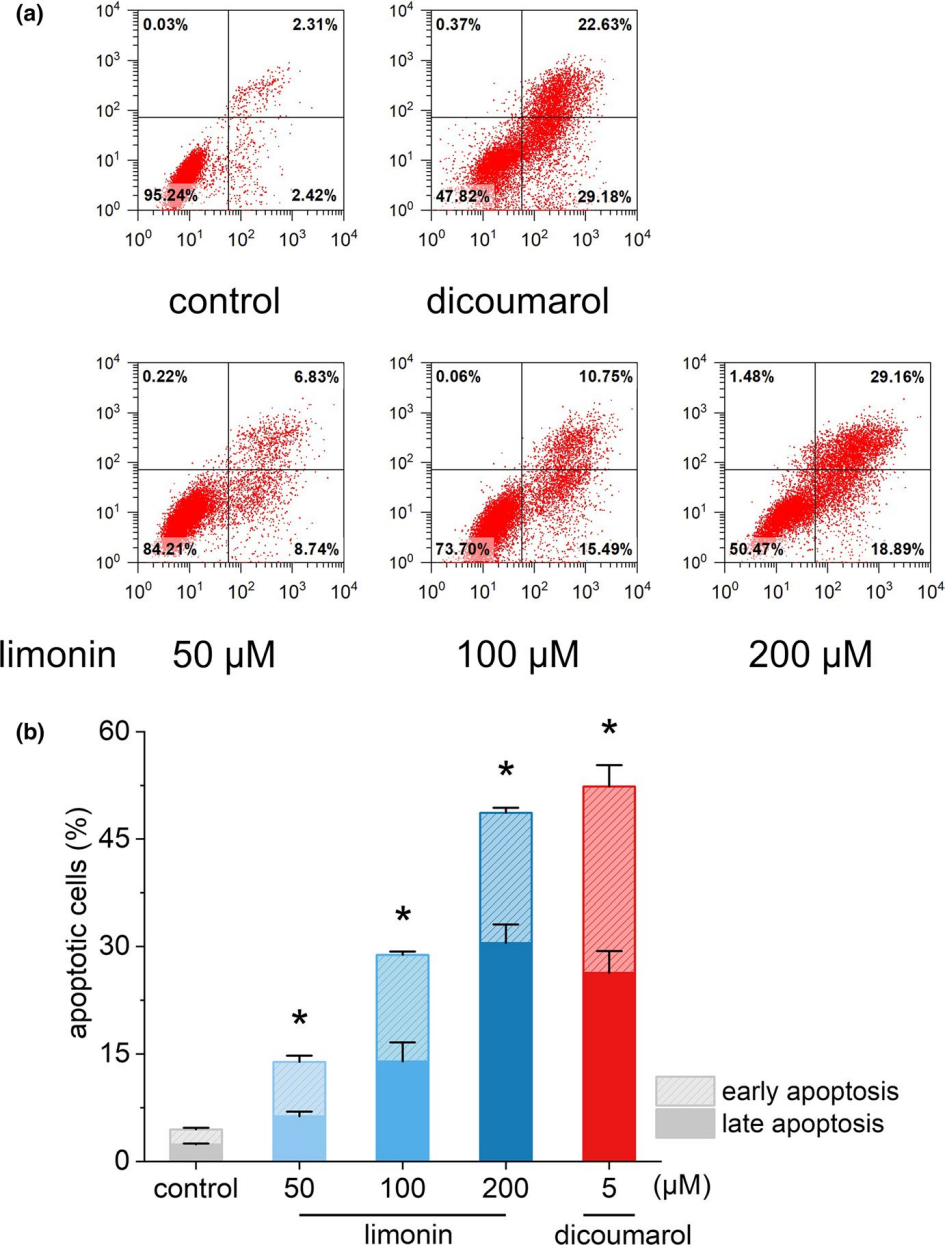
The effects of different concentrations of limonin on apoptosis of HL‐60 cells

Limonin was configured into solutions with final concentrations of 50, 100, and 200 μM, respectively, with the blank group as the control group and 5 μM dicoumarol (the specific inhibitor of NQO1) as the positive control. By analyzing the percentage of early and late apoptotic cells through flow cytometry, we evaluated the period during which limonin exerts a pro‐apoptotic effect on HL‐60 cells. Gratifyingly, limonin was found to cause a striking and concentration‐dependent increase in the levels of HL‐60 cells apoptosis (Figure 3b, *p* < .05). As can be seen from Figure 3a, the percentages of early and late apoptotic cells were indicated in the right lower and right upper quadrants, respectively. After treated with 50, 100, and 200 μM limonin, the mean percent early apoptosis of HL‐60 cells increased to 6.83%, 10.75%, and 29.16%, respectively, and the mean percent late apoptosis / necrosis also increased to 8.74%, 15.49%, and 18.89%, respectively, while those in the blank control group were only 2.42% and 2.31%, respectively. Moreover, the proportion of late apoptosis under 200 μM limonin even exceeded that of the positive control (22.63%). These results suggest that limonin plays a critical role in promoting apoptosis in HL‐60 cells, and its effect has a positive association with its concentration.

### 3.3 Regulatory activity of limonin on the expression of apoptosis‐related genes and proteins in HL‐60 cells

**FIGURE 4:**
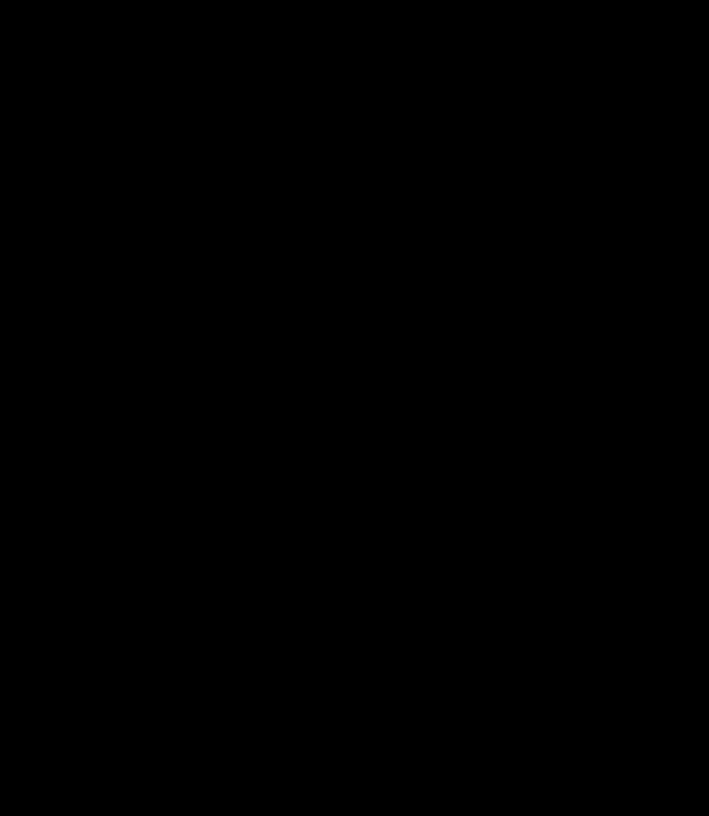
The effects of different concentrations of limonin on the expression of apoptosis‐related proteins in HL‐60 cells

**FIGURE 5:**
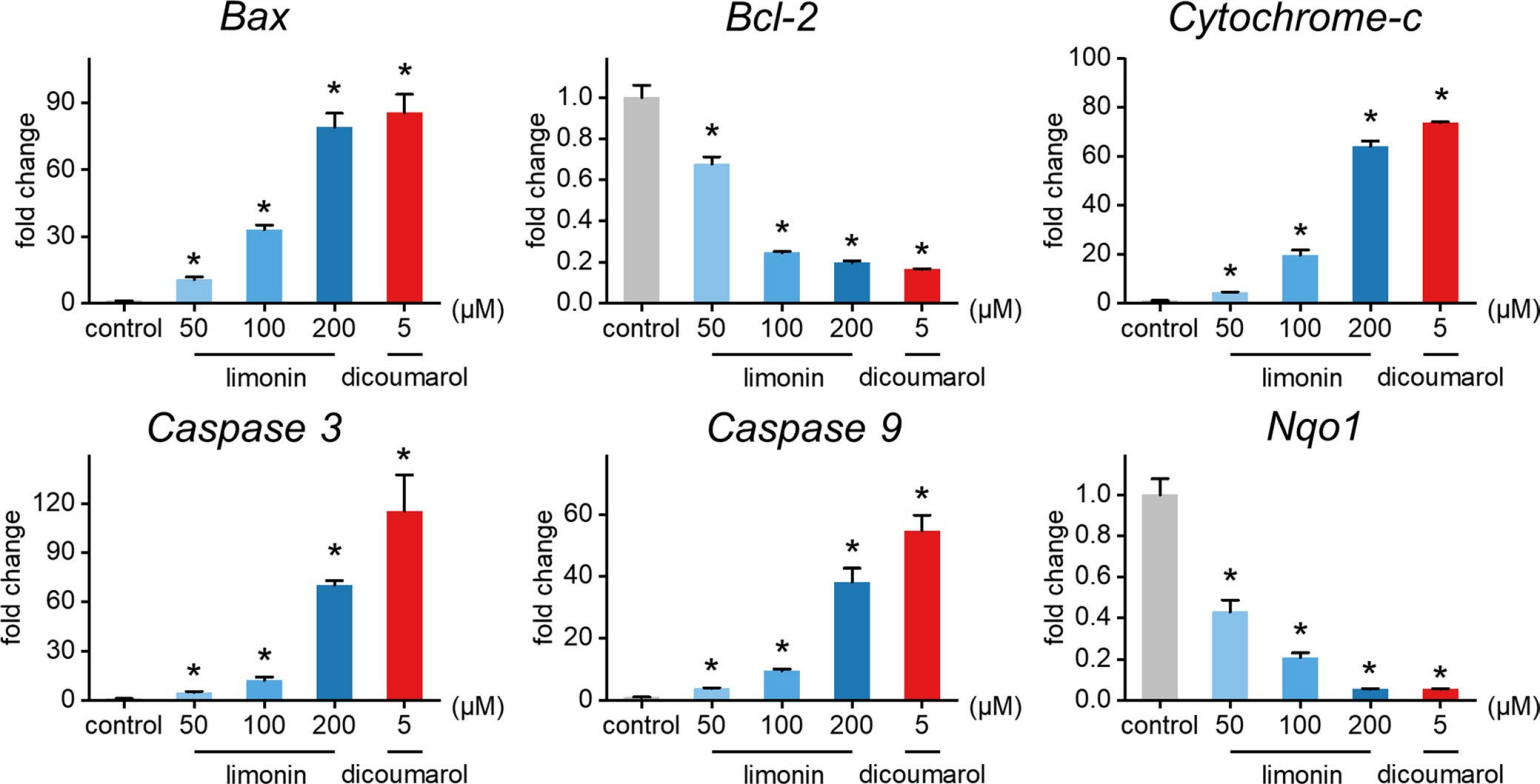
The effects of different concentrations of limonin on the expression of apoptosis‐related genes in HL‐60 cells

In order to investigate the molecular mechanism by which limonin promotes apoptosis of HL‐60 cells, we examined the expression levels of several key genes and corresponding proteins involved in the regulation of apoptosis as well as the NQO1 by Western blot. The results are shown in Figures 4 and 5. As shown in Figure 4a,b, NQO1 expression in HL‐60 cells was markedly down‐regulated compared with the control, and the higher the concentration of limonin, the more significant the down‐regulation effect. The results of qRT‐PCR also revealed that limonin significantly down‐regulated the expression of NQO1 with a dose‐dependent manner (Figure 5).

Apoptosis is a form of programmed cell death, which could be mediated by members of the Caspase family. One of the most classic apoptosis pathways is the intrinsic mitochondrial pathway. Bcl‐2 protein family play a crucial role in this pathway by regulating mitochondrial membrane potential. Both the anti‐apoptotic protein Bcl‐2 (B‐cell CLL/lymphoma 2) and the pro‐apoptotic protein Bax (Bcl2‐associated X protein) belong to the Bcl‐2 family (Huang et al., 2008; Wang et al., 2015). When the pro‐apoptotic protein Bax is transferred to the mitochondrial outer membrane to form a membrane channel, cytochrome c is released from the permeable mitochondrial membrane into the cytoplasm, binds with apoptosis activators to form an apoptotic complex, and then activates downstream Caspase9/3 signal cascades, which leads to apoptosis (Li et al., 2017; Ohtsuka et al., 2004; Repnik et al., 2012). Unique to this study, the relative expressions of key genes *Bax*, *Bcl‐2*, *cytochrome c*, *Caspase3*, *Caspase9* and key proteins Bax, Bcl‐2 cytochrome c, cleaved‐Caspase3 (activated form of Caspase3), cleaved‐Caspase9 (activated form of Caspase9), pro‐Caspase3 (inactive form of Caspase3), and pro‐Caspase9 (inactive form of Caspase9) were evaluated in the treatment of limonin with different concentrations. As depicted in Figures 4c,d, and 5, the relative gene expression of *Bax*, *cytochrome c*, *Caspase3*, *Caspase9*, and protein expressions of Bax, cytochrome c, cleaved‐Caspase3, and cleaved‐Caspase9 in the limonin experimental group were significantly up‐regulated, whereas the relative gene expression of *Bcl‐2* and protein expression of Bcl‐2 were significantly down‐regulated in a concentration‐dependent manner compared with the control group. There was no remarkable difference in the relative protein expression of pro‐Caspase3 and pro‐Caspase9 between treatments and control, except for the pro‐Caspase3 in 50 and 100 μM groups showed a predominant down‐regulation. On the basis of these results, we concluded that limonin can activate the endogenous apoptosis pathway mediated by mitochondria via up‐regulation of pro‐apoptotic proteins and down‐regulation of anti‐apoptotic proteins, thus inhibiting the proliferation and accelerating apoptosis of HL‐60 cells. This further proves that limonin has anti‐tumor potential from the molecular mechanism. Meanwhile, the down‐regulation of NQO1 expression by limonin indicates that limonin may indirectly act on the apoptosis pathway through regulating the expression activity of internal antioxidant enzyme, thereby exerting its inhibitory effect on tumor cells.

## 4 DISCUSSIONS

Limonin is an important secondary metabolite in citrus fruits, especially in seeds (Poulose et al., 2006). It has a variety of biological activities, such as anticancer, antioxidant, anti‐inflammatory, neuroprotective, and antibacterial. In recent years, a large number of studies have shown that limonin has great potential in cancer prevention and treatment by scavenging free radicals, stimulating the activity of glutathione transferase, inhibiting the activity of carcinogenic chemicals, and inhibiting the proliferation of tumor cells (Bodduluru et al., 2014; Galal et al., 2015, Gong et al., 2019). For example, Bae et al. (2020) found that limonin can induce cell apoptosis by activating p53 signal pathway, reducing the viability of SKOV‐3, A2780, and RMUG‐S ovarian cancer cells, and reversing the drug resistance of CISRSKOV‐3 cells by activating apoptosis. Gong et al. (2019) found that limonin can effectively inhibit lung cancer induced by benzopyrene (BP) in mice, and in vitro experiments showed that limonin can promote the production of reactive oxygen species (ROS) and induce apoptosis of A549 lung cancer cells. In addition, Berhow et al. (2000) found that limonin can inhibit the carcinogenicity of chemical carcinogens by inducing the activity of glutathione transferase (GST), and the effect is stronger in the stage of carcinogenesis. All the in vitro and in vivo researches have shown the intensive anticancer effect of limonin. In this study, through the functional study in vitro, we found that the proliferation of leukemia cell line (HL‐60) was significantly inhibited by limonin in a dose‐dependent manner by in vitro experiments. The results of flow cytometry showed that the toxicity of limonin on HL‐60 cells was achieved by inducing apoptosis. Western blot analysis further showed that limonin could activate the endogenous apoptosis pathway mediated by mitochondria, and accelerate apoptosis by up‐regulating the expression of pro‐apoptotic proteins (Bax, cytochrome c, cleaved‐Caspase3, and cleaved‐Caspase9) and down‐regulating the expression of anti‐apoptotic proteins Bcl‐2. These results were similar to the researches of Akihisa et al. (2013), Yao et al. (2018), and Chidambara Murthy et al. (2011), which further proved that limonin inhibited the growth of HL‐60 cells by inducing apoptosis through mitochondrial‐dependent pathway.

NQO1 is a flavoprotein that can catalyze two‐electron reduction of a broad range of substrates (Ross & Siegel, 2004). It has a variety of cytoprotective effects, especially in the prevention of cancer and oxidative stress‐related diseases (Oh & Park, 2015). However, in recent years, a large amount of evidence showed that NQO1 has a two‐sided effect in the occurrence of cancer. It can be used as both a tumor suppressor and a tumor promoter. It has been reported that *NQO1* knockout could inhibit the proliferation of glioblastoma cells, while overexpression promoted cell proliferation (Luo et al., 2018). In addition, the expression level of NQO1 has been found to be abnormally elevated in a variety of solid tumor cells (Oh & Park, 2015). Studies have shown that the overexpression of NQO1 was induced by overactivation of Nrf2, which helped malignant cells escape from extreme oxidative stress (Menegon et al., 2016). It can be seen that whether NQO1 plays an It can be seen that whether NQO1 plays an anti‐tumorigenic or tumorigenic effect depends on different conditions and cell types, which also reflects the potential of NQO1 as a target for cancer therapy. The research and development of innovative anticancer drugs have always been the focus of tumor research. Among them, natural products have received a lot of attention because of their low side effects and rich resources. At present, evidence has showed that natural products could exert their anticancer function by regulating the expression of NQO1 (up‐regulation or inhibition). For example, Zhao and Wu (2018) extracted a small molecule inhibitor of TACC3, spironolactone A (SpindlaconeA, SPL‐A), from dicoumarin. It was found that SPL‐A could promote TRAIL‐induced apoptosis of endometrial cancer cells by regulating apoptosis‐related proteins and inhibiting NQO1 activity in vitro. Lee et al. (2009) found that in HepG2 cells, TigloylgomisinH (TGH) isolated from *S*.*chinensis* significantly activated the gene expression mediated by antioxidant response element (ARE) through the nuclear accumulation of Nrf2, thus specifically inducing the activity of NQO1 to exert cancer prevention activity. It can be seen that natural products can indirectly play an anticancer role by regulating the activities of antioxidant enzymes. As an important bioactive compound in citrus fruit, limonin also shows many bioactivities by interacting with NQO1, such as anti‐inflammation. For example, limonin can up‐regulate the antioxidant pathway of Nrf2 activated by Sirt1 and increase the expression of its downstream target molecules HO‐1, NQO1, and GCLC/GCLM, so as to inhibit NF‐κB inflammation and reduce the hepatotoxicity induced by acetaminophen (APAP; Yang et al., 2020). Chen et al. (2017) also reported that limonin compound 7‐deacetylglucoside (7‐DGD) isolated from *Toona sinensis* fruit inhibited inflammation by activating Keap1/Nrf2/HO‐1 signal and up‐regulating the expression of antioxidant enzymes such as NQO1, HO‐1, and UGT1A1. However, how limonin and NQO1 interacted to play an anticancer effect has not been reported. Our research found that limonin accelerated the apoptosis of HL‐60 cells by regulating apoptosis‐related proteins and significantly down‐regulated the expression of antioxidant enzyme NQO1. It can be speculated that leukemic HL‐60 cells were easy to be in a hypoxic environment due to continuous and rapid proliferation, which could induce the overexpression of intracellular NQO1. The overexpression of NQO1 could help to clean up redundant ROS so as to reduce the damage of cancer cells. Limonin can weaken the protective effect by inhibiting the activity of NQO1, so that HL‐60 cells could not escape from oxidative stress and eventually undergo apoptosis. This provided new evidence and support for the exploration of limonin activity and the regulation of antioxidant enzyme activity of natural products.

Apoptosis refers to programmed cell death controlled by genes in order to maintain the stability of the internal environment. And mitochondrial‐dependent pathway is one of the main transmission pathways of apoptosis. cytochrome c (cytochrome c) in mitochondria is an important part of mitochondrial electron transport chain, and the release of it from mitochondria to cell matrix is a key step in the initiation of apoptosis (Haga et al., 2003; Wang & El‐Deiry, 2004). Bcl‐2 and Bax genes in the Bcl‐2 family are the most important regulators of apoptosis (Krueger et al., 2001). When the cells are internally stimulated, the homeostasis of the cellular environment is out of balance. Then, the mitochondrial membrane potential decreases, and Bax/Bcl‐2 proteins are recruited in the mitochondrial outer membrane, which can change the conformation of the mitochondria and form mitochondrial permeability transition pore (mPTP) on the mitochondrial outer membrane. The pro‐apoptotic factors such as cytochrome c are promoted to release into the matrix through mPTP, and cytochrome c binds to the apoptotic protease activator Apf‐1 to form a complex, inducing caspase‐9 precursor to cleave itself into active cleaved‐Caspase‐9 and further activate downstream cysteine (Caspase3, caspase‐7) cascade reaction, and finally induce apoptosis (Wang et al., 2018). Abnormal apoptosis is an important factor in cancer occurrence, so inducing tumor cell apoptosis has become an effective measure for cancer treatment (Kumar et al., 2013). Many studies have found that a lot of natural products can exert anticancer activity by inducing apoptosis of tumor cells (Wang, Zhong, et al., 2018). For example, Inonotus obliquus polysaccharides (IOP) could activate AMPK in a concentration‐depend manner and induce apoptosis of LLC1, A549‐LKB1 lung cancer cells while down‐regulating Bcl‐2, up‐regulating Bax protein expression, and enhancing the cleavage of Caspase3 and PARP (Jiang et al., 2020). Gambogic acid (GA) extracted from Teng Huang could trigger ROS‐mediated autophagy, then activated Caspase3 and downstream signal molecules such as NF‐kappa B, HIF‐1 α, AP‐1, and p53, and finally initiated the apoptosis pathway and lead to cell death. These results showed that (ROS) and caspase (Caspases) were potential mediators of cell death, and ROS (mainly produced by mitochondria) could activate Caspases‐3 protease activity and induce cell apoptosis or necrosis. Our study also found that limonin could reduce the activity of NQO1 antioxidant enzymes in a concentration‐dependent manner to weaken the cytoprotective effect, which may promoted the accumulation of intracellular ROS, then increased the proportion of Bax/Bcl‐2, induced the release of cytochrome c from mitochondria to the cytoplasm, and eventually activated Caspase3 and trigger apoptosis pathway. This result is very similar to the association of mitochondrial ROS, cytochrome c, and Caspase3 found by Suzuki et al (Suzuki et al., 1999) in N‐(4‐hydroxyphenyl) retinamide)‐induced apoptosis of cervical cancer cells.

Limonin belongs to triterpenes, and other triterpenoid natural products with similar structure also have important anticancer potential. For example, triterpenes isolated from Forsythia suspensa have obvious inhibitory effect on human digestive tract tumor cells, and the main way to induce apoptosis of SGC‐7901 cells may be to regulate the expression of apoptosis‐related proteins (Sun & Zhang, 2010). Triterpenes extracted from Pleurotus ferulae (ethylacetate fraction of Pleurotus ferulatus triterpenoid, PFTP‐E) can effectively induce apoptosis of Eca109 esophageal cancer cells, and its anti‐tumor mechanism is related to mitochondrial damage pathway, cycle arrest, and endoplasmic reticulum stress (Lei et al., 2019). Ursolic acid can regulate PI3K/AKT/mTOR signal pathway and hinder the development of colorectal cancer (Cui & Su, 2018). These studies show that the mechanism of triterpenoid natural products with different structures on cancer cells is different, and the action pathways of the same natural products on different cancer cells may also have variabilities, which provides a way to further explore the mechanism of limonin in anticancer and to explore the relationship between triterpene natural products with different structures and anticancer function.

## 5 CONCLUSION

In this study, by using Human promyelocytic leukemia cells (HL‐60), we investigated the pro‐apoptotic activity of limonin. Limonin inhibited the proliferation and promoted the apoptosis of HL‐60 cells in a dose‐dependent manner. Mechanism exploration demonstrated that the apoptosis of HL‐60 was related to the up‐regulation of Bax, cytochrome c, cleaved‐Casepase3, and cleaved‐Caspase 9 expression, and the down‐regulation of NQO1 and Bcl‐2 expression. The results showed that limonin may indirectly affect the apoptosis pathway by inhibiting the activity of antioxidant enzyme NQO1, thus promoting the apoptosis of HL‐60. Our study may complement the anticancer molecular mechanism of natural products.

## CONFLICT OF INTEREST

The authors declare that they do not have any conflict of interest.

## ETHICAL REVIEW

This study does not involve any human or animal testing.

## INFORMED CONSENT

Written informed consent was obtained from all study participants.

## Data Availability

Data sharing not applicable to this article as no datasets were generated or analysed during the current study.
